# Knowledge transfer in the field of parental mental illness: objectives, effective strategies, indicators of success, and sustainability

**DOI:** 10.1186/1752-4458-9-6

**Published:** 2015-01-28

**Authors:** Camilla Lauritzen, Charlotte Reedtz

**Affiliations:** Regional Centre for Child and Youth Mental Health & Child Welfare, UiT-Arctic University of Norway, Tromsø, Norway

**Keywords:** Health system improvement, Children of mentally ill parents, Effective strategies, Sustainability

## Abstract

**Background:**

Mental health problems are often transmitted from one generation to the next. However, transferring knowledge about interventions that reduce intergenerational transmission of disease to the field of parental mental illness has been very difficult. One of the most critical issues in mental health services research is the gap between what is generally known about effective treatment and what is provided to consumers in routine care.

**Discussion:**

In this article we discuss several aspects of knowledge transfer in the field of parental mental illness. Effective strategies and implementation prerequisites are explored, and we also discuss indicators of success and sustainability.

**Summary:**

Altogether, this article presents a rationale for the importance of preventive strategies for children of mentally ill parents. Furthermore, the discussion shows how complex it is to change clinical practice.

## Background

Knowledge transfer can be defined as: The process which one unit – for example; an individual, a group, a department or an organization – is affected by the experience of another [1]. It is important to highlight that providing information, presenting facts, arranging informative courses or even giving lectures is not the same as knowledge transfer. This is because knowledge alone is not necessarily sufficient in order to create behavior change. In essence, knowledge transfer is about facilitating behavior change. One way of explaining knowledge transfer is to regard it as the process of organizations seeking to improve performance by implementing a new practice [1, 2].

How is knowledge transferred from one unit or organization to another? There are several factors that can facilitate or impede knowledge transfer in organizations and it is definitely possible to design organizations and procedures to promote knowledge transfer [1, 2]. This is however a very complex area, consisting of many important mechanisms. The literature is extensive on this field, and we will discuss the most important mechanisms of knowledge transfer later in this article, but for now let’s just agree that there are many issues to address if you want to understand the mechanisms of knowledge transfer.

### The field of parental mental illness

Many studies have documented that mental illness is very common [3]. Mental illness is defined as a psychological pattern, potentially reflected in behavior, that is generally associated with distress or disability and is not considered part of normal development [4]. According to the DSM IV criteria, the term mental disorder refers to a clinically significant behavioral or psychological syndrome or pattern that occurs in an individual, is associated with present distress or disability and represents a manifestation of a behavioral, psychological, or biological dysfunction in the individual. The most common mental health problems are anxiety, depression and substance abuse issues [5].

In a 2009 report on mental illness in Norway, The Norwegian Institute of Public Health (2009) estimated that up to 50% of the population will suffer from mental health problems at some point during their lifetime [5].

Adults with mental health problems are not less likely to be parents then the rest of the population [6]. Several international studies the past two decades have indicated that children with mentally ill parents are at risk of developing mental health problems themselves [7–9]. Parental mental illness is considered a powerful risk-factor, with a potential of serious impact for the children. For instance: parents with depression have more difficulties in interaction with their children, are more intrusive, less involved and less responsive [10–12].

More than one third of these children develop serious and long-lasting problems. Early in life, these children run a higher risk of abuse and neglect, depression, eating disorders, conduct problems and academic failure. Later in life, they are at a higher risk of depression, anxiety disorders, substance abuse, eating problems and personality disorders [13–15].

Maternal symptoms of anxiety and depression increased the risk of emotional and disruptive problem behaviors in children as early as 18 months of age, according to new research findings from the Norwegian Institute of Public Health. And these problems are often found to be long lasting [16].

It is especially when Parental mental illness is present during the early years of life that it triggers dys-regulated emotion patterns, negative emotionality and insecure attachment. A lot of documentation exists on the serious effects parental mental illness may have on the early developmental stages of a child’s life [17–22]. It is safe to say that early intervention is essential to counteract permanent damage to the child’s developmental path.

Parental mental illness may interrupt the neurological development in offspring [23, 24]. Since the brain is not fully developed when we are born, the experiences a child has growing up will have direct effect on the development of the brain [23, 24]. Children of mentally ill parents are in many cases exposed to traumatic childhood experiences, for example: they can be witnesses to violence, or they may have been subject to abuse or neglect. This is commonly referred to as developmental traumas. Developmental traumas result from growing up in a context of ongoing danger, maltreatment, unpredictability, and/or neglect. Developmental traumas tend to surface as several disorders, i.e., regulatory disorder during infancy, attachment disorders, hyperkinetic conduct disorder at school age, or combined conduct and emotional disorders during adolescence [23, 24]. Children that live under stressful conditions over time, will produce a lot of stress hormones and the child is in a way becoming programmed into a state of constant emergency preparedness. The child’s cognitive resources are tied up in being in a state of emergency, and this delays and impairs the child’s development in other areas [23, 24].

Regulatory competence is a key concept. Emotional regulation is developed early in life in interaction with caregivers. Emotional regulation is a complex process involving: the subjective experience (feelings), cognitive responses (thoughts), physiological responses (for example heart rate or hormonal activity), and behavior (such as bodily actions or expressions) [25]. Children who have been neglected or abused have been found to have a dysfunctional self-regulatory competence [23].

The impact parental mental illness may have on offspring is commonly ignored within the adult mental health services [26], even though there is thorough documentation that Parental mental illness is a powerful risk factor for children. The objective of including a focus on the patient’s children is linked to prevention, because there are measures that can be taken to counteract the risk, for instance by implementing a prevention perspective in adult mental health services. There is a substantial amount of research documenting that teaching parents positive parenting strategies to promote children’s self-confidence, pro-social behaviors, problem-solving skills and academic success reduces the risk for those children [27, 28]. There is also growing evidence to support the idea that strengthening protective factors for children of mentally ill parents may reduce the incidence or prevalence of some mental disorders [4]. There are several well-known protective factors for children of mentally ill parents, and they are commonly divided in three categories: family related factors (such as parental participation in the child’s life, sensitive upbringing strategies and consistent child-rearing approaches), individual factors (gender, self-esteem, intellectual capacity, social skills), and structural factors (positive school environment, social network, socio-economic status) [21].

The prevention objective is threefold. First of all it’s about preventing children from developing poor regulatory competence, insecure and disorganized attachment [23].

It also involves preventing added burden to the parents disease, because research has documented that treatment alone is not as effective as when it is combined with family focused strategies [29]. And thirdly, and hopefully as a result of this; preventing mental illness from being transmitted from one generation to the next [10].

## Discussion

There are several important aspects to discuss in terms of successfully implementing a child perspective within adult mental health services. Prevention work is generally difficult, and so is implementation work.

### Effective strategies

When we discuss preventive strategies and early intervention approaches, it is important to investigate what kind of evidence we have that prevention is effective. Durlak and colleagues conducted a meta-analysis in 1997, demonstrating that programs to prevent mental disorders can be effective for children [30]. In 2002, Jané-Llopis found that effects of prevention programs are stable over time, and are effective for populations with different levels of risk [31]. So generally there is evidence to support the use of preventive programs.

What about the programs specifically developed for the field of parental mental illness? Many of the strategies within preventive interventions involve aspects of parent training. The idea is that parent training programs can help families and children to regulate the child’s thoughts, feelings and behavior [32]. Within the field of parenting there are several programs that have an extensive evidence base [33]. Parenting programs may be used to promote good mental health in children also in the field of parental mental illness [34]. Parent training programs is a good option for some of the families affected by parental mental illness, however depending on the diagnosis and the severity of the situation. Furthermore, these programs are used by a growing number of local communities, and may be easier to get access to than programs that are more specifically designed for parental mental illness issues.

There are also some programs that are more specifically designed to target families affected by parental mental illness. In 2012 a meta-analysis was published. The authors assessed the evidence in terms of effectiveness of the preventive interventions in decreasing the risk of mental disorders in the offspring of mentally ill parents.

The conclusion in this meta-analysis is that the evidence indicated that such interventions may be effective and that different approaches to treatment of the families may be equally effective [35]. However, the results from the studies reported in the meta-analysis mainly consisted of mothers with affective disorders and depression, and the results may therefore be less applicable to parents with other mental disorders and to fathers. Additionally, several studies included were of questionable standards and this may have led to an overestimate of the effects [35]. The authors do however point to the need for further studies of sufficient size and high methodological quality.

In 2012 a review of intervention programs for children whose parents have a mental illness was published, providing an **overview** of available interventions. The authors of this review divided the interventions in three groups:

1. Family intervention programs.
2. Peer-support programs for children.
3. Online interventions for children/adolescents whose parents have a mental illness.

The most common component in the programs was provision of psychosocial education about mental illness. Only some of the interventions had been evaluated, and very few had been evaluated in Randomized Controlled Trials.

The authors concluded that more evaluations are needed in this field, and particularly studies that incorporate validated outcome measures [36].

So in the field of parental mental illness, what would be effective strategies for knowledge transfer? An effective strategy should take into account the fact that parental mental illness has serious consequences for children and that we can prevent the trans-generational transmission of mental illness by preventive interventions.

Furthermore, there are several existing interventions with good evidence of effect that can be used to train parents in better parenting strategies; e.g., the Incredible Years program or the PMTO (parent management training Oregon) intervention. There is a problem when policy makers and other agencies decide to disseminate programs that have no documented effects. In worst case scenarios, programs may prove to have negative effects. And even if the situation should be that the strategy chosen had no effects - it would be a major waste of resources. This is why programs that have been evaluated and found to be effective should be priority number one, if such documentation exists. When planning effective strategies in this context, evidence based programs are preferable to interventions without evidence of effect. However, in order for a strategy to be effective, the implementation aspect has to be a part of the equation.

### Implementation of effective strategies

Knowledge transfer can be challenging and one perspective that may be useful in addressing these challenges is to be found in the substantial body of implementation literature. The essence of implementation is behavior change. Implementation is defined as *a specified set of activities designed to put into practice an activity or program of known dimensions* [37].

Currently, little is known about the processes required to effectively implement evidence-based programs on an international scale. Rigorous research to support the implementation activities that are being used is even scarcer. A major goal in the Implementation Research area is to help establish an evidence base for the implementation processes [37].

Implementation may involve different connotations for different people. When referring to implementation, different agents refer to a variety of contrasting activities and strategies; and the strategies they refer to represent varied depth and dedication [38]. The differing views of implementation may be categorized as degrees of implementation in the following way. The first degree is Paper implementation. This refers to putting new policies and procedures into place; e.g. legislation, commission documents and guidelines. However, changing policies and procedures does not change practice in itself.

The second degree is called Process implementation. This means incorporating new procedures into an organization; i.e. providing new guidelines and supervision, and changing reporting forms, among other things. However, the “mechanism” to change may not exist because this strategy does not incorporate any tools or specific intervention to guide the change in behavior. The highest degree of implementation is commonly referred to as Performance implementation. This is the most extensive degree of implementation, meaning that it provides content and tools to practitioners so that new procedures and processes have functional components for change. According to the implementation research literature, performance degree implementation strategies are more likely to be successful than the other two degrees of implementation [38].

There are several core components that work together in any attempt to implement and sustain effective innovations [39]. These core components are; decision support data system (for instance organizational fidelity measures), a facilitative administration that provides leadership and support in the process, system intervention to ensure the availability of financial, organizational and human resources, recruitment and selection, pre-service training, consultation and coaching, and finally staff performance evaluations. The integrated and compensatory nature of the core components embodies the perspective that organizations are dynamic, and there will be variations in the relative contributions of each component to the overall outcomes. However, if the core components are not taken into account and assessed in implementation projects, the result may be unsuccessful implementation processes [39]. Even though there is some evidence to support the importance of the core components [39], more rigorous implementation research should be conducted to extend the evidence base of core implementation components.

### Behavior change

Implementation of new routines involves behavior change. Many strongly believe that increasing knowledge and changing attitudes also change people’s behavior. This is linked to a belief that awareness campaigns, education and a general focus on a subject, will cause behavior change in people. In the study of changing clinical practice to safeguard children of mentally ill parents, this view implies that information and courses for health professionals should have the potential to change clinical practice. Within health promotion campaigns, this has been a particularly common strategy [40], for instance campaigns to encourage people to stop smoking. The no-smoking strategy has been effective because the strategy has been multi-layered; from restricting the availability, banning smoking in public areas to strategies to change attitudes, and strategies to help people gain control over their behavior.

The point is: in order for a strategy to improve the situation for families affected with parental mental illness, the strategy must incorporate more than information about risk-factors. Behavior change is complicated. This implies that is not sufficient to simply point out why something should change, how the changes are to come about must also be determined. There is no reason to expect that positive general attitudes to improved services for children of mentally ill – or even increased knowledge about the risk of these children - automatically will change clinical practice. There is no theoretical or empirical foundation to expect specific skills and behaviors to arise from a general dissemination of knowledge and positive attitudes.

### Where should we begin?

It is not of indifference where a process of knowledge transfer or behavior change should begin. A model which was developed by Maybery and Reupert in 2009 was designed as a hierarchy of points of intervention to affect workforce change, because it is unlikely that higher level activities can be successful unless the lower levels of the hierarchy already exists in the organization [41].

The lowest level represents the importance of the policies within an organization, for example guidelines. Strategies to change practice have to be embedded in the organization, and the management has to be on board with the aims to change.

The next level of the hierarchy consists of issues relating to the workforce for example Workers’ attitudes, skills and knowledge. The most important areas for workers to develop in this context include reporting systems, assessment, referral procedures and psycho-education in regard to the service user. The groundwork of stage one and two will then enable the workers to engage with the service user.

Level three of the hierarchy represents the barriers families themselves bring in. Parents may not know the consequences their illness has on their children, or they may be reluctant to discuss this with others. Parents may have fears that discussing their insecurity and problems in childrearing may lead health personnel to worry about the quality of care their children receive, and consequently that others will report them to the Child Protection authorities. Once organizational anchoring has been done and the workforce has been trained to engage with the clients in a family oriented perspective, only then is it realistic to achieve a clinical practice that incorporates the parental mental illness aspect.

You have to have the bottom levels first in order to achieve the top level. This means that in the process of changing clinical practice, one should always start with initial groundwork such as; creating a detailed protocol that accounts for time and resources within the organization, assessing organizational needs, addressing requirements from the health authorities and so on. According to the model, the resistance and unwillingness the service users may have to discuss their children will be less prevalent when organizational issues and workforce related problems have been addressed.

We did a slight modification of the model to adapt the model to a Norwegian context [42]. We believe that an infinite amount of resources and efforts at the lower level will not allow movement upwards, because the movement is hindered by a contextual dimension. We therefore added a contextual level to the model, to incorporate these challenges. The added dimension encompasses two important aspects that are external conditions, but with a potential large impact on the movement from one stage to the next in the model. The first aspect is 1) the organization of mental health care services, Services for adults and services for children are two very different organizations and not necessarily co-operating. The second aspect is 2) the geographical context in which the mental health care services are provided. Sometimes the home-community is very far away from the hospital the adult is admitted to. This makes it difficult, if not impossible, to bring in the children to visit and receive preventive interventions within adult mental health services. A possible solution to this could be to offer interventions in the local communities instead of the hospitals. However, since the workforce at the hospitals have better knowledge of the mental health issues of the parents; perhaps telecommunication solutions could be explored?

### Indicators of success

Sometimes it is difficult to know for certain that the strategies chosen have been effective. What we think may be the case is not necessarily accurate. Clinicians or managers may have a hunch that what is done within the clinic to support children of mentally ill is good, based on perhaps one person’s *very* dedicated work in the area. It does not always mean that *everyone* is doing dedicated work. We need reliable ways of assessing success.

In terms of measuring success, we are talking about two different processes.

**Figure 1:**
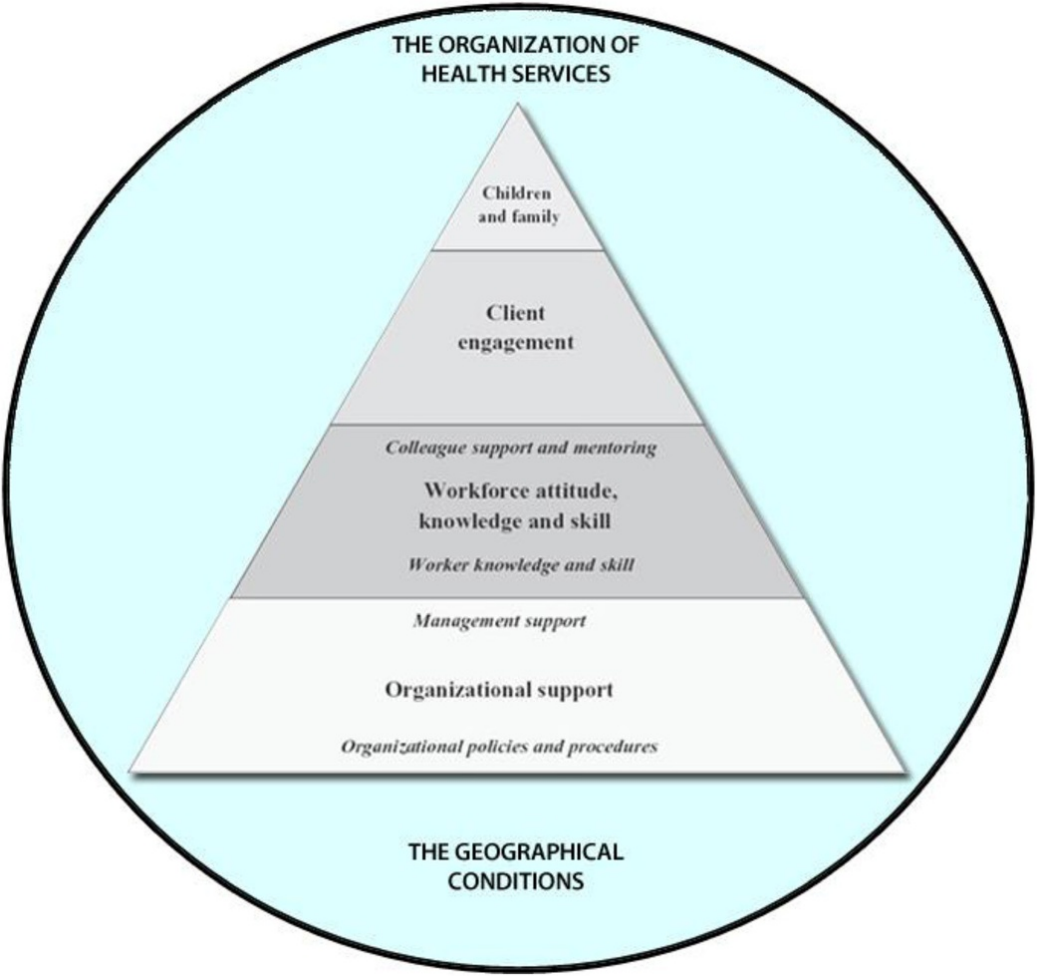
**Adapted hierarchy to affect workforce change (Maybery & Reupert 2009, Adapted by Lauritzen & Reedtz).**

We’re talking about evaluating the effects of the interventions and in that sense monitoring if the strategy is successful (levels 3 and 4 in the hierarchy, Figure 1). We’re also talking about monitoring the implementation process and keeping an eye on the process of change at all times (levels 1 and 2 in the hierarchy, Figure 1).

Indicators of success in terms of client engagement and services for children and families (level 3 and 4, Figure 1) can be detected by studying the effects of the interventions. This implies that in terms of the interventions one chooses to apply in the field of parental mental illness, one way of measuring success is to look at the outcomes for children and families. To look for indicators of success you have to look into the evaluations on the intervention’s effect on parents and children, in efficacy studies, effectiveness studies or other approaches to evaluation. Good outcomes for children is in itself an indicator of a successful approach. Monitoring the outcomes for children is important in addition to monitoring the process of implementation of the intervention in real life. Fidelity is of course also very important. In the field of program evaluation, the term fidelity denotes how closely a set of procedures were implemented as they were supposed to have been. For example, it’s difficult to draw conclusions from a study about effective strategies in the field of parental mental illness if the practitioners are not able or willing to follow the procedures they received in training. Subsequently, higher fidelity is correlated with better outcomes, and therefore a significant factor in the assessment of success indicators. Studies that used fidelity scales have found better outcomes for consumers when services adhere closely to an approach with specified critical components and standards [43].

The other approach to measuring success is linked to studying the process of implementation, and documenting activities related to level 1 and 2 in the hierarchy (see Figure 1). In Implementation research – measuring processes of change is crucial in order to keep track of the indicators of success, and one aspect that is important to address is readiness to change. Organizational readiness to change is considered a critical precursor to achieve successful implementation of complex changes in healthcare settings. This implies that the implementation strategies should encompass activities to create motivation to change. On-going assessment of organizational readiness is very important in order to be successful in any attempts to change.

Furthermore, to keep track of the process it is important to evaluate the core variables that you want to change in the implementation strategy. In our study, these have been linked to knowledge, attitudes, collaborative routines and clinical practice related to families with parental mental illness. The road to success may not be as straight forward as we imagine when we set up our protocols and project plans, which is why we need to monitor the process. We need to be aware of what is going on along the way.

An example of a tool that can be used to monitor the process of change is measuring collective efficacy. The term collective efficacy refers to individual group members’ perceptions of the capability of the group to achieve specific goals [44]. In therapeutic organizations it represents the practitioners’ and the leaders’ perceptions as a whole that their agency is capable of creating positive outcomes for the children.

The readiness of an organization for successful implementation of evidence-based practices may be predicted in part by an organization’s level of collective efficacy [45]. This means that a valid measure of collective efficacy in services may be particularly interesting in implementation research. However, as a self-report measure of capability to create positive outcomes for patients and families the tool is subjective and therefore limited. A solution to this limitation could be to include more concrete measures such as case load.

### Sustainability

If the implementation process is successful, and we have successfully transferred knowledge about parental mental illness and about effective interventions to achieve the objectives of better outcomes for parents and children; how do we get it to stick? In terms of new-practice glue, the term to discuss this is sustainability. Sustainability addresses the issue of how the new practice, the transferred knowledge, is to survive in the every-day practice [46].

Finances are also a big issue, as many preventive interventions fail to become sustainable because insufficient resources are provided. Cost-benefit analyses play an important role in the planning and decision making process of implementation projects, and sustainability issues need to be a part of the analyses.

The goal with sustainability is the long term survival and continued effectiveness of the implementation site in the context of a changing world. A review article published in 2012 by Stirman and colleagues provides an overview of the current state of the research literature on the sustainment of interventions [46]. One finding in this review was that partial sustainability was very common, meaning that elements of the implementation had survived, but not necessarily all elements that make up a program package.

The studies that reported on full sustainability were few and did not include long-term reports of post-implementation outcomes. Follow up measures to monitor sustainability is necessary and preferably more than just one-year follow up studies.

The conclusion was that the body of literature on sustainability was fragmented and underdeveloped [46]. To advance what is known about sustainability will require time, resources and funding. Appropriate planning assessment and allocation of funds would result in much better understanding of why and how some interventions last and others do not.

There are of course a lot of challenges related to sustaining interventions in the field of practice. The sustainability strategies should encompass strategic support within the organization. The success and sustainability of evidence based practices can be substantially influenced by the quality of organizational support systems for the program and leadership support.

It is important to retain an ongoing capacity for sustaining the interventions.

Implementation projects need to be properly anchored in the organization. The management must actively support the implementation of a new practice, and this should be reflected in the policies within an organization, such as guidelines, service statements, protocols and interagency guidelines. Sufficient human resources and time to take on the new tasks must be allocated. Additionally, the managers must emphasize that the new practice is relevant and worth taking on. Otherwise, the hope of establishing the new routines within practice as usual is at risk. There must be ongoing recruitment of practitioners to carry out the interventions, which implies resource allocations. Sustaining interventions is reliant on core implementation personnel, but also on-going routine evaluations to monitor the implementation activities.

## Conclusion

To sum up, where do we stand in general on knowledge transfer in the field of parental mental illness? We know something about effective strategies, we have well defined objectives, we have a few effective interventions, and especially interventions that target parenting behavior have a good evidence base. The evidence base on interventions specifically designed to address families affected by parental mental illness is growing, but more studies should be conducted in this area. We have models to help us understand behavior change and complex implementation issues. We even have ways to measure indicators of success, and we know something about how to create sustainable practices. The question is perhaps: do we have the patience? We need to recognize that knowledge transfer or implementation work is time consuming.

On the one hand; Researchers need to acknowledge the fact that they might have to work closer with the field of practice, and perhaps invest in longer time perspectives than traditional research projects. On the other hand; practitioners need to commit to the project protocols and invest time in adopting the new routines. If we pull together we can perhaps succeed in the endeavor to bridge the gap between research and practice.

Incorporating effective strategies in adult mental health services can potentially prevent parental mental illness being transmitted from one generation to the next.

It is therefore important for both researchers and practitioners to remember why the extensive strategies to change clinical practice are important. For the children and families who may benefit from the changes, it may mean a world of difference.

### Ethics statement

The study this debate is based on has been considered by the Regional Committees for Ethics in Medical Research. The project was approved by the data protection officer (DPO) who has approved of the total protocol for this project.

### Data sharing statement

No additional data available.
